# Biomarkers to assess the risk of bladder cancer in patients presenting with haematuria are gender-specific

**DOI:** 10.3389/fonc.2022.1009014

**Published:** 2022-09-23

**Authors:** Brian Duggan, Declan O’Rourke, Neil Anderson, Cherith N. Reid, Joanne Watt, Hugh O’Kane, Ruth Boyd, David Curry, Mark Evans, Michael Stevenson, Mary Jo Kurth, John V. Lamont, Peter Fitzgerald, Mark W. Ruddock

**Affiliations:** ^1^ South Eastern Health and Social Care Trust, Ulster Hospital Dundonald, Belfast, United Kingdom; ^2^ Belfast Health and Social Care Trust, Belfast City Hospital, Belfast, United Kingdom; ^3^ Randox Laboratories Ltd, Randox Science Park, Antrim, United Kingdom; ^4^ Northern Ireland Clinical Trials Network, Belfast City Hospital, Belfast, United Kingdom; ^5^ Department of Epidemiology and Public Health, Queens University Belfast, Belfast, United Kingdom

**Keywords:** biomarkers, bladder cancer, cytology, cystoscopy, diagnostic, HABIO, haematuria triage, gender

## Abstract

**Introduction:**

Haematuria is a common red flag symptom of urinary tract cancer. Bladder cancer (BC) is the most common cancer to present with haematuria. Women presenting with haematuria are often underdiagnosed. Currently, no gender-specific tests are utilized in clinical practice. Considerable healthcare resources are needed to investigate causes of haematuria and this study was set up to help identify markers of BC. The aim of the study was to define biomarker algorithms in haematuria patients using an expanded panel of biomarkers to diagnose BC and investigate if the algorithms are gender-specific.

**Materials and Methods:**

A total of n=675 patients with a history of haematuria were recruited from Northern Ireland hospitals. Patients were collected on a 2:1 ratio, non-BC (control) n=474: BC n=201. A detailed clinical history, urine and blood samples were collected. Biomarkers, known to be involved in the pathobiology underlying bladder carcinogenesis were investigated. Biomarkers differentially expressed between groups were investigated using Wilcoxon rank sum and linear regression.

**Results:**

Biomarkers were gender specific. Two biomarker-algorithms were identified to triage haematuria patients; male – u_NSE, s_PAI-1/tPA, u_midkine, u_NGAL, u_MMP-9/TIMP-1 and s_prolactin (u=urine; s=serum); sensitivity 71.8%, specificity 72.8%; AUROC 0.795; and female urine biomarkers - IL-12p70, IL-13, midkine and clusterin; sensitivity 83.7%, specificity 79.7%; AUROC 0.865. Addition of the clinical variable infection to both algorithms increased both AUROC to 0.822 (DeLong p=0.014) and to 0.923 (DeLong p=0.004) for males and females, respectively. Combining clinical risk factors with biomarker algorithms would enable application of the algorithms to triage haematuria patients.

**Conclusion:**

Using gender-specific biomarker algorithms in combination with clinical risks that are associated with BC would allow clinicians to better manage haematuria patients and potentially reduce underdiagnosis in females. In this study, we demonstrate, for the first time, that blood and urine biomarkers are gender-specific when assessing risk of BC in patients who present with blood in their urine. Combining biomarker data with clinical factors could improve triage when referring patients for further investigations.

## Introduction

Lack of proper investigation of haematuria, especially in females, is a relevant clinical problem. On the other hand, haematuria investigation can be a considerable burden within healthcare systems. However, the risk of haematuria being a manifestation of underlying disease varies with age, gender and type of haematuria (microscopic *vs*. macroscopic). Blood in the urine can be alarming, however, in many instances the cause is benign. The most common cause for haematuria is infection. Microscopic haematuria is estimated to occur in up to 20% of asymptomatic men over 60 years of age (1). However, monosymptomatic macroscopic haematuria is a key symptom that should be investigated in all cases.

Although there are many causes for blood in the urine, those most commonly identified include bladder infection, stones in the kidneys or bladder, benign prostate enlargement (BPE), cancers of the bladder, kidney or prostate, and in many instances, no cause of bleeding is found. Strenuous activity may cause episodic haematuria. Benign discolouration of the urine can also be caused by dyes, foods, menstruation, and drugs (e.g., rifampacin). However, if doubt exists, referral for investigations is mandatory.

BC is one of the most significant causes of haematuria. BC is the tenth most common cancer worldwide; the sixth most common cancer in men and the seventeenth most commonly occurring cancer in women (2). **Table 1** describes the estimated number of new cases of BC and mortality rate in 2020 worldwide, Europe, and North America.

**Table 1:** Bladder cancer incidence and mortality.

https://gco.iarc.fr/today/home		Estimated number of new cases in 2020	Estimated number of deaths in 2020	Mortality Rate (%)	Crude Mortality Rate (per 100,000)	ASR (World) (per 100,000)
**Worldwide**	All	573,278	212,536	37.1	2.7	1.9
	Male	440,864	158,785	36.0	4.0	3.3
	Female	132,414	53,751	40.6	1.4	0.9
**Europe**	All	203,983	67,289	33.0	9.0	3.0
	Male	156,658	50,816	32.4	14.1	5.5
	Female	47,325	16,473	34.8	4.3	1.2
**North America**	All	89,997	21,045	23.4	5.7	2.1
	Male	69,080	15,129	21.9	8.3	3.5
	Female	20,917	5,916	28.3	3.2	1.1

Estimated number of new cases of bladder cancer 2020, worldwide, Europe and North America.

https://gco.iarc.fr/today/.

ASR, age-standardised rates.

BC is three times more common in males than females (3). However, studies have shown that females have higher stage of BC at diagnosis (4, 5). In many cases, there are significant delays in diagnosing BC in women who present with haematuria (6). Furthermore, women have been shown to have poorer survival outcomes when adjusted for all stages (5). Therefore, women need to be given special diagnostic consideration when presenting with haematuria (7, 8).

The exact cause of BC is unknown. However, risk factors include smoking, chronic bladder inflammation, occupational exposure to chemicals, radiotherapy to the pelvic area and chemotherapy (5, 9). The overall risk of BC is 20.4% in patients presenting with monosymptomatic haematuria, although this percentage can be dramatically higher when stratifying patients for age, risk factors, exposure, and gender (10). Cystoscopy with the adjunct of urine cytology is the gold standard for BC investigations. White light cystoscopy is known to miss small multifocal tumours and carcinoma *in situ*. However, better visualization of tumours has been achieved using new optical technologies e.g., blue-light cystoscopy and narrow-band imaging (11, 12). Studies have shown that cancer survival, including BC is significantly better if detected and treated at an early stage (13–15). Thus, there is an urgency to correctly diagnose BC in patients presenting with haematuria. However, cystoscopy is a costly invasive procedure which presents some risks, including infection, bleeding and patient discomfort (16). CT urography is needed for upper urinary tract investigation. However, clinical guidelines vary globally on which patients should be investigated, and what these investigations should entail (17).

Depending on country, BC costs from diagnosis to death between $89,287 and $202,203 per person (2009) (18). In 2012, the total European Union healthcare cost for BC was €4.9 billion accounting for 5% of the total cancer health expenditure (19). In the US, BC has the highest costs per person with treatments costing an estimated $3.4 billion annually with $2.9 billion in direct treatment-related costs (2009). The lifetime cost per non-muscle invasive BC patient was estimated at between $96,000 to $187,000 in the US (20). Thus, it is important for healthcare systems to improve efficiency by reducing unnecessary invasive investigations in haematuria patients without compromising diagnostic accuracy (10).

Previously, we investigated whether single biomarkers and/or multivariate algorithms could assist with prediction of BC in patients presenting with haematuria (21). This study demonstrated that biomarkers representing diverse pathways can significantly improve the AUROC statistic based on demographics. Twenty-three biomarkers were investigated.

The current Haematuria Biomarker study (HABIO) aim was to define algorithms in a larger cohort of haematuria patients using an expanded panel of biomarkers to diagnose BC and investigate if the algorithms are gender-specific. We investigated urine and serum biomarkers known to be involved in the pathobiology underlying bladder carcinogenesis. Identification of a biomarker algorithm could be used to triage haematuria patients in primary care into low and high-risk categories. Patients identified at low risk would be managed in primary care, thus reducing the number of referrals for invasive and costly procedures.

## Materials and methods

### HABIO patients

**Figure 1:**
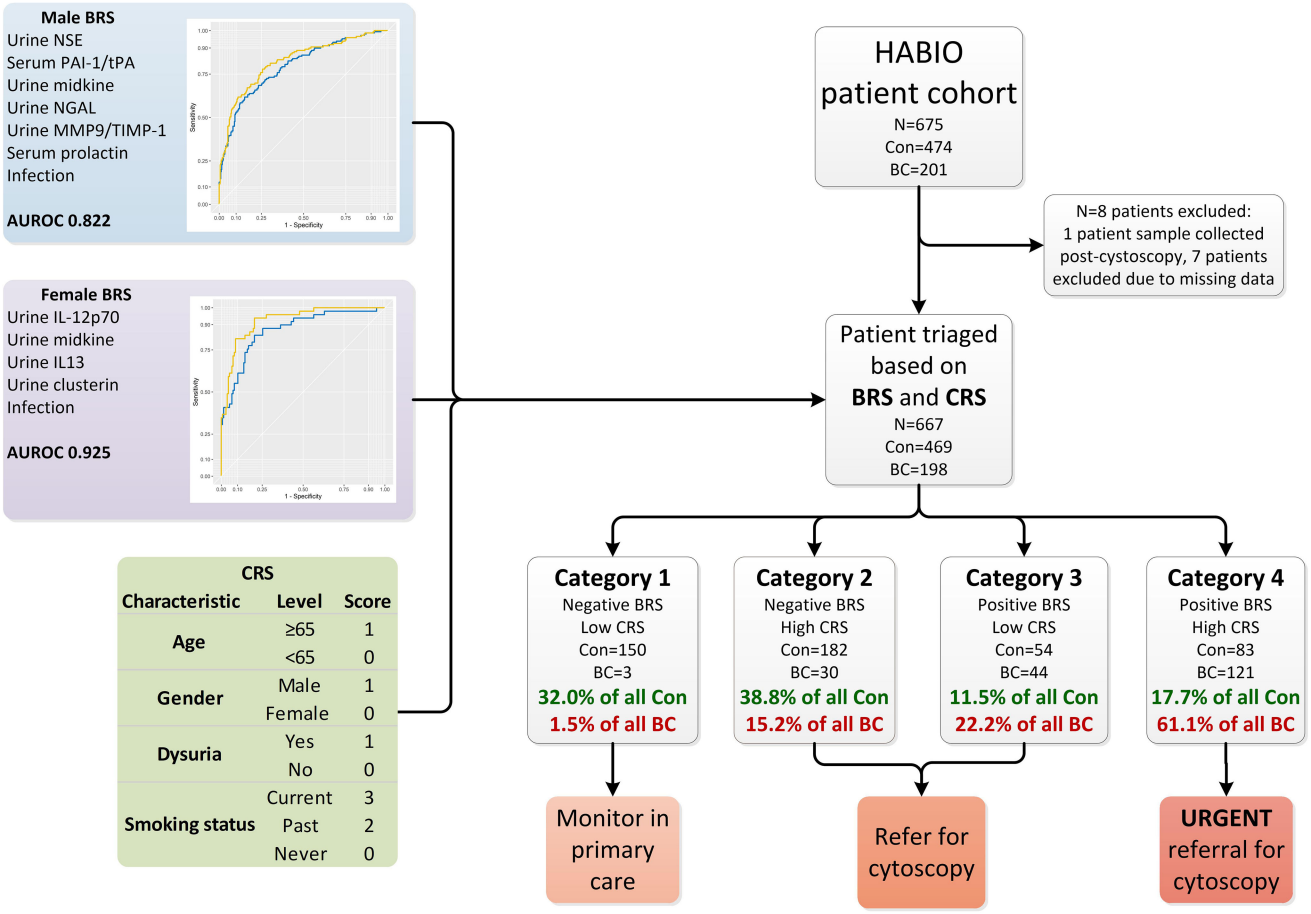
Overview of haematuria triage system. The HABIO patient cohort was triaged based on the combination of results from the BRS and CRS into four categories. The biomarkers that contributed to the BRS for males included urine NSE, serum PAI-1/tPA, urine midkine, urine NGAL, urine MMP-9/TIMP-1 and serum prolactin (blue line AUROC 0.795) and with urothelial infection (yellow line AUROC 0.822) is presented in the top left. The biomarkers that contributed to the BRS for females included urine IL-12p70, midkine, IL-13 and clusterin (blue line AUROC 0.865) and with urothelial infection (yellow line AUROC 0.925) is presented in middle left. The CRS is presented in the table bottom left. Con, control; BC, bladder cancer; BRS, biomarker risk score; CRS, clinical risk score; NSE, neuron specific enolase; PAI-1/tPA, plasminogen activator inhibitor-1/tissue plasminogen activator; NGAL, neutrophil gelatinase-associated lipocalin; MMP-9/TIMP-1, matrix metallopeptidase-9/tissue inhibitor metallopeptidase-1; AUROC, area under receiver operating curve; IL-12p70, interleukin-12p70; IL-13, interleukin-13.

Patients (N=675) presenting with haematuria (microscopic haematuria n=247, macroscopic haematuria n=428) and undergoing cystoscopy were recruited to the HABIO case-control study between 2013 and 2016 at Northern Ireland hospitals - Ulster, Craigavon and Belfast City (**Figure 1**). Of the patients presenting with macroscopic haematuria 194/428 (45.3%) did not have any other symptoms associated with BC (such as loss of bladder control, dysuria, or increased frequency of urination (day or night)).

Research nurses (RNs) collected and recorded demographic, clinicopathological data and information about treatments on a Recruitment Form. The patient was asked about their lifestyle, hobbies and pastimes, urinary symptoms, occupation(s), current medication(s) and whether they have ever been exposed to hazardous chemicals. Weight, height, and blood pressure were recorded. RNs collected and recorded investigation results for the Final Review Form.

All BC cases (N=201) ≥40 and ≤80 years were recruited with pathologically proven (newly diagnosed (n=146, 72.6%) or recurrent (n=55, 27.4%)) BC at pre-assessment clinics, from inpatients or at planned cystoscopy clinics. A consultant pathologist undertook a review of diagnostic pathology for all patients. Pathological grade and stage and presence/absence of inflammatory infiltrate together with other relevant information were recorded. A consultant cytopathologist undertook a review of diagnostic cytology and recorded diagnosis and noted the presence/absence of inflammatory cells. Cytology was assessed on Papanicolaou and Giemsa-stained preparations. Urinary infection was diagnosed based on patient clinical history, biomarkers and dipstick analysis.

Control patients (N=474; infection (n=221 (male, n= 148, female, n=73); BPE (n=213); healthy (n=30); no diagnosis (n=119); other cancers (n=3); other benign conditions (n=24); prostate cancer (n=10)) (**Table 2**) were recruited from haematuria clinics following negative cystoscopy and negative findings from BC investigations. The incidence of infection was lower in male controls (n=148/334 (44.3%)) compared to female controls (n=73/140 (52.1%)). Written informed consent was obtained from all patients and samples were collected in the outpatient setting. HABIO inclusion and exclusion criteria are described in **Supplementary 1**.

**Table 2:** HABIO patient demographics and behaviours.

Demographics	Control	Bladder Cancer	p value
Age	65.2 ± 10.0 (n=474)	68.1 ± 9.2 (n=201)	<0.001
Gender (Male)	334/474 (70.5%)	151/201 (75.1%)	0.218
Ethnicity (White Caucasian)	470/474 (99.2%)	199/201 (99.0%)	0.848
BMI	29.0 ± 5.0 (n=474)	28.8 ± 5.7 (n=201)	0.367
Systolic Blood Pressure	135.6 ± 19.0 (n=474)	135.1 ± 16.4 (n=201)	0.855
Diastolic Blood Pressure	77.7 ± 10.9 (n=474)	77.9 ± 11.4 (n=201)	0.908
Hypertension (yes)	310/474 (65.4%)	130/201 (64.7%)	0.857
Haematuria (macroscopic)	254/474 (53.6%)	174/201 (86.6%)	<0.001
Smoking (pack years)	29.3 ± 33.4 (n=263)	37.7 ± 35.6 (n=151)	<0.001
Total tar exposure (kg)	3.06 ± 3.29 (n=263)	4.38 ± 4.30 (n=151)	<0.001
Smoker (Current and Past)	263/474 (55.5%)	151/201 (75.1%)	<0.001
Alcohol consumption (≤ 4 units pw)	268/474 (56.5%)	112/201 (55.7%)	0.845
Alcohol consumption (> 4 units pw)	206/474 (43.5%)	89/201 (44.3%)	0.845
Loss of bladder control (male)	82/333 (24.6%)	27/151 (17.9%)	0.100
Loss of bladder control (female)	74/140 (52.9%)	13/50 (26.0%)	0.001
BPE/BPH	210/334 (62.9%)	41/151 (27.2%)	<0.001
Infection	221/474 (46.6%)	33/201 (16.4%)	<0.001
UTI treated sole cause of haematuria	44/221(19.9%)		
No diagnosis	109/474 (23%)		
Diabetes	74/474 (15.6%)	37/201 (18.4%)	0.370
Other benign diagnosis	24/474 (5.1%)	3/201 (1.5%)	0.030
Prostate Cancer	13/474 (2.7%)	12/201 (6%)	0.042
Newly diagnosed TCC		146/201 (72.6%)	
Recurrent TCC		55/201 (27.4%)	

Data are presented as mean ± SD or number/total (percentage).

BMI, body mass index; BPE/BPH, benign prostate enlargement/hyperplasia; UTI, urinary tract infection; TCC, transitional cell carcinoma.

The study was approved by the Office for Research Ethics Committees Northern Ireland (ORECNI 11/NI/0164), and R&D Offices of participating hospitals and registered with ISRCTN (ISRCTN25823942) and Cancer Research UK public trial database. The study was conducted according to Standards for Reporting of Diagnostic Accuracy (STARD) (22).

### HABIO patient samples

Patient urine (25 ml) and blood (35 ml) samples were collected and processed by a RN/technician. Whole blood was allowed to clot, and the resulting serum was removed and aliquoted. Unfiltered and uncentrifuged urine samples were immediately aliquoted and frozen at -80°C. Frozen samples were transported on dry ice to Randox Laboratories Ltd, Crumlin, UK. Urine and serum samples were thawed on ice and centrifuged (1200 x g, 10 minutes, 4°C) to remove particulate matter prior to analysis.

### HABIO biomarker measurements

Patient samples were run in triplicate at Randox Clinical Laboratory Services (RCLS), Antrim, UK. Randox scientists were blinded to patient data. Results are expressed as mean ± SD. Details of biomarker limits of detection (LOD)/mean detectable dose (MDD) (ELISAs) and analysers are described in Supplementary 2. Data below the LOD/MDD was inputted as 90% of the LOD/MDD (23).

### Statistical analyses

Statistical analyses were undertaken using IBM SPSSv25 and R (24). Continuous clinical characteristics and biomarker data were analysed using Wilcoxon mean rank sum and descriptive characteristics were analysed using Chi-Squared contingency test to identify which factors were differentially expressed between control and BC. Statistical significance was taken at the p<0.05 level and results are presented as mean ± SD where appropriate. Biomarkers differentially expressed between groups were investigated by forward and backward Wald binary logistic regression and least absolute shrinkage and selection operator (Lasso) to identify algorithms to diagnose BC. Comparisons between AUROC were undertaken using the DeLong test.

## Results

Demographic and clinical information for recruited HABIO participants are described in **Table 2**. BC patients were older and were more likely to present with macroscopic haematuria, thus there is a clinical need to stratify patients and identify high-risk groups. Newly diagnosed and recurrent BC were combined into one group. The male to female ratio for haematuria was 2.6:1.0 and for BC was 3.0:1.0. HABIO patients with BC smoked more and had higher total tar exposure. Loss of bladder control was noted for both males and females however, this was only significant for females. Alcohol consumption was not significantly different between groups.

### Medications

Statins and PPIs were the most common medication classes. However, there was no difference in the mean number of medications taken by either control or BC patients (5.2 ± 3.8 *vs*. 4.9 ± 3.7, p=0.462, respectively). Anticoagulants (e.g., aspirin) were also not significantly different between groups (control 137/474 (28.9%) *vs*. BC 54/201 (26.9%), p=0.657).

### Point of care assays and investigations

NMP22 was positive for G2 (n=11/109 (10%)) and G3 (n=28/81 (35%)) BCs and failed to detect any G1 tumours (n=0/6). Three urinary markers were identified by auction dipstick as significantly different between control and BC; namely, protein (p<0.001), specific gravity (p=0.029) and blood (p<0.001).

### HABIO biomarker measurements

Urine and serum biomarker results are described in **Supplementary 3** and **4**, respectively. It was noted that biomarkers were gender-specific. Thus, separate gender-specific biomarker algorithms were identified.

### Male BC biomarker algorithm

The following biomarker combination was identified for the detection of BC in males: urine NSE, serum PAI-1/tPA, urine midkine, urine NGAL, urine MMP-9/TIMP-1 and serum prolactin: AUROC 0.795 (95%CI 0.751-0.839) (sensitivity 71.8%, specificity 72.8%). Including urinary infection in the biomarker algorithm increased the AUROC to 0.822 (95%CI 0.780-0.865) (sensitivity 77.9%, specificity 74.3%) (DeLong p=0.014) (**Figure 1**).

### Female BC biomarker algorithm

The following urinary biomarker combination was identified for the detection of BC in females: IL-12p70, IL-13, midkine and clusterin: AUROC 0.865 (95%CI 0.806-0.924) (sensitivity 83.7%, specificity 80.4%). Including urinary infection in the biomarker algorithm increased the AUROC to 0.925 (95%CI 0.885-0.965) (sensitivity 81.6%, specificity 91.3%) (DeLong p=0.004) (**Figure 1**).

### Biomarker risk score

Biomarker combination algorithm(s) can be applied clinically to determine if a patient is at risk of having BC. Patients with a score ≥ the value of the set point (cut-off) would be positive (at risk of having BC), whereas patients below the cut-off would be negative. The biomarker risk score (BRS) for BC was calculated using the male and female algorithms (**Figure 1**, male and female BRS panels).

### Clinical risk score

Clinical risk score (CRS) for BC was calculated for each HABIO patient. The CRS is a cumulative score utilising clinical and demographic measurements, namely, age, gender, dysuria, and smoking status (**Figure 1**, CRS panel). These clinical factors were most relevant to the HABIO cohort. Setting defined cut-offs of ≤3 for low CRS and >3 for high CRS, based on achieving the highest negative predictive value (NPV) (**Table 3**), enabled stratification of the haematuria patients into low or high-risk groups. For example, a 60-year-old male, without dysuria, non-smoker would score: 0 (age) + 1 (gender) + 0 (dysuria) + 0 (smoking status) = 1 (low CRS). Whereas, a 70-year old female, without dysuria, smoker would score: 1 (age) + 0 (gender) + 0 (dysuria) + 3 (smoking status) = 4 (high CRS).

**Table 3:** Positive and negative predictive value by biomarker risk score (BRS) and clinical risk score (CRS) by gender.

	% PPV (95% CI)	% NPV (95% CI)
CRS	36.5 (31.9 - 41.3)	81.4 (76.1 - 86.0)
Male BRS	52.6 (45.9 - 59.2)	88.8 (84.2 - 92.4)
Male BRS and CRS	37.7 (32.9 - 42.7)	97.8 (92.2 - 99.7)
Female BRS	61.1 (48.9 - 72.4)	95.7 (90.1 - 98.6)
Female BRS and CRS	38.7 (30.1 - 47.9)	98.4 (91.5 – 100.0)
CRS and BRS	38.1 (33.9 - 42.4)	98.0 (94.4 - 99.6)

The NPV and PPV were calculated for risk scores comparing category 1 to categories 2, 3 and 4. To maximise NPV a CRS cut-off of <3 was selected.

PPV, positive predictive value; NPV, negative predictive value; CI, confidence interval; CRS, clinical risk score; BRS, biomarker risk score.

### Clinical performance

Patients with a negative BRS and low CRS (category 1) could be monitored in primary care if patients agreed with that approach. Patients with a high CRS (category 2) or positive BRS (category 3) could be referred for further investigations. However, patients who present with a positive BRS and high CRS (category 4) would automatically be referred for urgent investigations.

Testing the BRS and CRS in the HABIO cohort (**Figure 1**) resulted in 153 patients being assigned to category 1. Of these, 150 patients (22.5% of the total cohort) were correctly classified. Three patients (0.4% of the total cohort) with BC were misclassified. The three patients that were misclassified had low grade BC (pTaG2) (**Table 4**).

**Table 4:** Bladder cancer stage and grade for each category.

		Category			
		**1**	**2**	**3**	**4**
**Stage**	**pTa**	3	23	30	74
	**pT1**		2	10	24
	**pT2**			3	11
	**pT3**			1	6
	**pT4**				2
	**Not known**		5		4
**Grade**	**G1**			4	6
	**G2**	3	21	27	65
	**G3**		5	13	45
	**Not known**		4		5

Low risk patients were designated pTaG1/G2 disease.

pT, primary tumour; G, grade.

Using the BRS and CRS, 37.4% of all BC patients were triaged into categories 2 and 3. The treatment pathway for patients assigned to category 2 or 3 would require referral for further investigations, including cystoscopy. Over 70% of control patients were categorised into categories 1 and 2 (332/474 (70.8%)).

Over 61% (121/198) of patients assigned to category 4 had BC (18.1% of total cohort). Category 4 represents the highest patient risk group. The stage and grade for all BCs by category are shown in **Table 4**.

## Discussion

Patients presenting with unexplained haematuria in primary care are frequently referred to secondary care for investigations. The challenge for clinicians is to triage patients who present with haematuria in both primary and secondary care. Clinicians are faced daily with patients who present with a range of symptoms and use their skills to assess malignancy risk. In addition to triaging those requiring further investigations, it would be advantageous to healthcare systems to reduce the number of investigations. Furthermore, the COVID-19 pandemic has placed significant pressure on healthcare services globally and there is a desire to limit the number of individuals being referred to hospital reducing exposure to COVID-19 (10).

Cystoscopy is the gold standard for detecting BC. However, cystoscopy is invasive and not without risk. As such, new non-invasive urine-based tests are currently under development for BC detection e.g., EpiCheck (25), Bladder CARE (26), DNA methylation (27). However, none of these tests have demonstrated efficacy based on gender.

BC has a high recurrence rate. Therefore, patients require lifetime monitoring. As such, BC has the highest treatment costs for all cancers. Thus, genomic screening to identify oncogenic markers that are prognostic and gender-specific would allow a personalised approach in patient management (28, 29).

The HABIO study was based on a previous study (21). The aim of HABIO was to define algorithms in a larger cohort of haematuria patients using an expanded panel of biomarkers to diagnose BC. Urine and serum biomarkers known to be involved in the pathobiology underlying bladder carcinogenesis were investigated. Identification of an algorithm with biomarkers could be used to triage haematuria patients into low and high-risk categories. This study of n=675 patients demonstrated that BC patients were significantly older than controls and more likely to present with macroscopic haematuria. It is known that BC is a disease of the elderly (30). Macroscopic haematuria, male sex, and age over 60 years have been identified as independent risk factors for BC (31, 32). The ratio of male to female for BC was 3:1 consistent with other reports. However, the male to female ratio for BC can vary from 6:1 to 2:1 depending on geographical location, cultural and social issues, and the number of women with haematuria who are submitted to invasive testing by cystoscopy (4, 33).

Biomarkers known to be involved in the pathobiology of bladder carcinogenesis, were investigated in urine (43 biomarkers) and serum (32 biomarkers) from the HABIO cohort. Individually, none of the biomarkers achieved the sensitivity or specificity required to replace cystoscopy. Furthermore, both FDA-approved biomarkers, BTA and NMP22, did not perform well in the HABIO patient cohort (AUROC 0.661 and 0.579, respectively) and were considerably lower than previously published reports (34–39). Our patient cohort was primarily Caucasian. Furthermore, many of the patients presented with low grade BC.

From the results, a high proportion of biomarkers were identified as gender-specific. Therefore, the data was separated by gender and algorithms were developed that could stratify risk of BC in both male and female haematuria patients.

The following combination of biomarkers was identified for detection of BC in males: urine NSE, serum PAI-1/tPA, urine midkine, urine NGAL, urine MMP-9/TIMP-1 and serum prolactin (AUROC 0.795, sensitivity 71.8%, specificity 72.8%, NPV 85.2% and PPV 54.3%) (**Figure 1**). Urothelial infection was demonstrated to be an important risk factor and when added to the male algorithm the AUROC increased to 0.822 (DeLong p=0.014).

The best combination of biomarkers for the detection of BC in females included urine IL-12p70, IL-13, midkine and clusterin (AUROC 0.865, sensitivity 83.7%, specificity 80.1%, NPV 93.3% and PPV 60.3%) (**Figure 1**). Similarly, inclusion of urothelial infection in the female algorithm increased the AUROC to 0.925 (DeLong p=0.004). Female HABIO patients who presented with infection were unlikely to have BC. However, a small number of female haematuria patients (3/50 (6.0%)) had both an infection and BC. Therefore, any infection should be treated before the biomarker algorithm is applied. The AUROC for the urinary biomarker combination for females was higher than that found in males. Furthermore, for males, both serum and urine biomarkers were required in the algorithm. Males presented with more comorbidities e.g., BPE. Adding clinical risk e.g., infection, significantly improved the performance of the biomarker algorithm(s). In females, addition of infection to the biomarker algorithm increased the specificity from 80.1% to 91.3%. Therefore, the algorithm could potentially be used to rule out BC and reduce the number of unnecessary cystoscopies in females.

To apply the biomarker algorithm clinically a similar approach was used to that described previously (23, 40). Results generated from the gender-specific algorithms were converted into a biomarker risk score (BRS) and combined with four clinical factors (age, gender, dysuria, smoking status) in the form of a clinical risk score (CRS). Application of the results from the BRS and CRS combination triaged the haematuria patients into four categories: category 1 low risk to category 4 high risk of BC (**Figure 1**). Thus, if a patient was triaged into category 1, they could be monitored if there was patient agreement. Although patients from categories 2 to 4 are referred for cystoscopy, only category 4 patients would be considered the most urgent.

In this study, using both the BRS and CRS, 153/667 (22.9%) haematuria patients were triaged as low risk. Three BC patients were in this category however, they were low risk BC (3/153 (2.0%)) (**Table 4**). Therefore, application of this triaging system clinically could potentially reduce the number of referrals of haematuria patients to secondary care by >20%.

This triaging system grouped 204 haematuria patients as category 4, the highest risk category. Of these 121/204 (59.3%) had BC. Therefore, applying this triage system identified the main proportion of BC patients. These individuals would be fast tracked for urological investigations.

Of the 212 patients in category 2, 30/212 (14.2%) had BC; this represents 15.2% of all BC. Of the 98 patients allocated to category 3, 44/98 (44.9%) had BC; this represents 22.2% of all BC. The highest risk categories were 3 and 4 where the haematuria patients had a positive BRS. Application of the triage system correctly categorised 83.3% (165/198) of BC patients into categories 3 and 4. HABIO identified two gender-specific algorithms (i) male - urine NSE, serum PAI-1/tPA, urine midkine, urine NGAL, urine MMP-9/TIMP-1 and serum prolactin, and (ii) female - urine IL-12p70, IL-13, midkine and clusterin.

## Conclusion

Differentiating which haematuria patients require further invasive investigation is challenging and clinically risky. BC can be a highly lethal disease, especially in high-risk non-muscle invasive or muscle-invasive cases. Moreover, the clinical problem of underdiagnosis in women presenting with haematuria or infection and haematuria and underlying BC is a crucial one. The proposed gender-specific algorithms can help to solve both clinical problems mentioned above. The accuracy of the algorithms can be improved, achieving a very high specificity in women, when using them in combination with other clinical parameters. For the first time, a gender-specific approach is proposed to triage haematuria patients, potentially able to accurately identify BC male and female patients and send them for further invasive investigation, reducing unnecessary procedures and hospital costs.

## Data availability statement

The raw data supporting the conclusions of this article will be made available by the authors, without undue reservation.

## Ethics statement

The study was approved by the Office for Research Ethics Committees Northern Ireland (ORECNI 11/NI/0164) and R&D Offices of participating hospitals. The patients/participants provided their written informed consent to participate in this study.

## Author contributions

The authors confirm contribution to the paper as follows: study conception and design: BD, DO’R, CR, HO’K, MS, JL, PF and MR. Data collection: BD, DO’R, NA, CR, HO’K, RB, DC, ME and MR. Analysis and interpretation of results: BD, DO’R, NA, CR, JW, HO’K, ME, MS and MR. Draft preparation: BD, CR, JW, MK, JL, PF and MR. All authors contributed to the article and approved the submitted version.

## Funding

The HABIO study was part funded by a grant from Invest Northern Ireland (RD0412515).

## Acknowledgments

The authors would like to acknowledge the patients, patient representatives and the clinical research nurses who were involved in the study. This work was supported by the Northern Ireland Cancer Trials Network and Belfast Experimental Cancer Medicine Centre.

## Conflict of interest

CR, JW, MK, JL and MR are paid employees of Randox Laboratories Ltd but hold no shares in the company. PF is the Managing Director and owner of Randox Laboratories Ltd, a privately-owned company.

The remaining authors declare that the research was conducted in the absence of any commercial or financial relationships that could be construed as a potential conflict of interest.

## Publisher’s note

All claims expressed in this article are solely those of the authors and do not necessarily represent those of their affiliated organizations, or those of the publisher, the editors and the reviewers. Any product that may be evaluated in this article, or claim that may be made by its manufacturer, is not guaranteed or endorsed by the publisher.
