# Supplementary material for: Biomarkers to assess the risk of bladder cancer in patients presenting with haematuria are gender-specific
Source: Front Oncol. 2022 Sep 23;12:1009014. doi: 10.3389/fonc.2022.1009014 (PMC9539269; doi:10.3389/fonc.2022.1009014)
Supplement: Supplementary file 1 [file DataSheet_1.docx]

**Supplementary 1**

**Inclusion and Exclusion Criteria**

***Bladder cancer patients***

- Written informed consent to participate in the study
- Aged between 40 and 80 years
- Current haematuria or a history of haematuria
- Cystoscopy within the last 6 months or planned cystoscopy
- No chemo- or radio- therapy in the three weeks prior to recruitment
- No previous history of cancers other than bladder cancer
- Suspicion of bladder cancer or proven bladder cancer

***Control patients***

- Written informed consent to participate in the study
- No previous history of cancer
- Of the same gender, approximate age, and smoking status (where possible) to a bladder cancer patient already recruited to HABIO
- Current haematuria or a history of haematuria
- Negative cystoscopy within the last 3 months, but at least 48h after the procedure
- No chemo- or radio- therapy in the three weeks prior to recruitment

***Exclusion Criteria***

***Bladder cancer patients:***

- No written informed consent to participate in the study
- Aged < 40 or > 85 years
- No history of haematuria
- No recent or planned cystoscopy
- Chemo- or radio- therapy in the three weeks prior to recruitment
- Previous history of cancer(s), other than bladder cancer
- No suspicion of bladder cancer or proven bladder cancer

***Control patients***

- No written informed consent to participate in the study
- Previous history of any cancer
- Not of the same gender, approximate age and smoking status of a patients already recruited as a bladder cancer patient
- No history of haematuria
- No recent or planned cystoscopy
- Chemo- or radio- therapy in the three weeks prior to recruitment

**Supplementary 2 Biomarker Description**

Biochip Array Technology (BAT) was used by Randox Clinical Laboratory Services (RCLS), Randox Science Park, Antrim, UK by scientists blinded to patient data, for the simultaneous detection of multiple biomarkers from a single patient sample [1]. The analytical sensitivity of the biochip(s) was as follows: cystatin C 0.60 ng/ml; EGF 2.5 pg/ml; IFNγ 2.1 pg/ml; IL-2 4.8 pg/ml; IL-2Ra 0.12 ng/ml; IL-23 13.0 pg/ml; IL-3 8.78 pg/ml; IL-4 6.6 pg/ml; IL-6 1.2 pg/ml; IL-6R 0.62 ng/ml; IL-7 1.11 pg/ml; IL-8 7.9 pg/ml; IL-10 1.1 pg/ml; IL-12p70 2.61 pg/ml; IL-13 5.23 pg/ml; VEGF 14.6 pg/ml; TNFα 4.4 pg/ml; IL-1α 0.8 pg/ml; IL-1β 1.6 pg/ml; MCP-1 13.2 pg/ml; NSE 0.26 ng/ml; NGAL 17.8 ng/ml; sTNFR1 0.24 ng/ml; D-dimer 2.1 ng/ml; sTNFR2 0.2 ng/ml; and CRP 0.67 mg/ml. Functional sensitivity for CEA and PSA (free and total) on the biochip were 0.29, 0.02 and 0.45 ng/ml, respectively. All biochips were run on an Evidence Investigator analyser according to manufacturer’s instructions (Randox Laboratories Ltd, Crumlin, UK). The analytical sensitivity for HDL, LDL and cholesterol were as follows: direct HDL cholesterol (HDL) 0.189 mmol/l (7.30 mg/dl), direct LDL cholesterol (LDL) 0.189 mmol/l (7.30 mg/dl) and cholesterol 0.865 mmol/l (33.4 mg/dl), respectively. HDL, LDL and cholesterol were run on a Daytona analyser (Randox Laboratories Ltd, Crumlin, UK). The analytical sensitivity for urinary microalbumin was 5.11 mg/l. Microalbumin was analysed on a Daytona Plus analyser (Randox, Crumlin, UK). The analytical sensitivity for prolactin was 6.52 mIU/l. Prolactin was run on an Evidence Evolution analyser (Randox, Crumlin, UK). The analytical sensitivity for cystatin C was 0.4 mg/l. Serum Cystatin C was run on a Daytona analyser (RCLS, Antrim, UK). Triglycerides were run on a Daytona analyser (RCLS, Antrim, UK). Creatinine (μmol/l) measurements were performed by Randox Testing Services, Crumlin, UK, using a quantitative *in vitro* diagnostic assay from Randox (Crumlin, UK) on a Daytona analyser, according to manufacturers’ instructions (Randox). The creatinine assay is linear up to 66,000 μmol/l and has a lower sensitivity of 311 μmol/l.

**Commercial ELISA kits**

The following biomarkers were detected using commercially available ELISA kits, as per manufactures instructions; all patient samples were run in triplicate: 8-hydroxy 2 deoxyguanosine (8OHdG), MDD 0.1 ng/ml (Cell Biolabs, San Diego, US); Bladder tumour antigen (BTA), MDD 0.65 U/ml (Polymedco, New York, US); Cluster of differentiation 44 (CD44), MDD <0.113 ng/ml (Abcam, Cambridge, UK); UBC II (CK-8, CK-18), MDD 0.1 ng/ml (IDL, Bromma, Sweden); Cytokeratin-20, MDD 0.1 ng/ml (CK-20) (BlueGene, Shanghai, China); Clusterin, MDD 0.189 ng/ml (R&D Systems, Abingdon, UK); CXCL16, MDD 0.007 ng/ml (R&D Systems, Abingdon, UK); Cystatin B, MDD 0.013 ng/ml (R&D Systems, Abingdon, UK); Epithelial growth factor (EGF), MDD 25 pg/ml (Randox, Antrim, UK); Fatty acid-binding protein – adipose (FABP-A), MDD 0.05 ng/ml (Biovendor, Abingdon, UK); Tumour necrosis factor receptor superfamily member 6 (FAS), 5 pg/ml (RayBio, Georgia, US); Hyaluronic acid (HAD), MDD 0.1 U/l (MyBioSource, San Diego, US); C-X-C ligand 1 motif/growth regulated alpha protein (CXCL1/GROα) MDD 10 pg/ml (R&D Systems, Abingdon, UK); Interleukin 18 (IL-18), MDD 42.8 pg/ml (Randox, Antrim, UK); LIM and SH3 domain (LASP-1), MDD 6.25 pg/ml (Cusabio, Houston, US); Muscle type-2 pyruvate kinase (M2-PK), MDD 3.4 ng/ml (Randox, Antrim, UK); Caspase-cleaved CK-18 fragments (M30), MDD 20 U/L (Previva, Paudex, Switzerland); Midkine, MDD 8 pg/ml (CellMid, Sydney, Australia); Matrix metallopeptidase 9/Neutrophil gelatinase-associated lipocalin complex (MMP-9/NGAL), MDD 0.013 ng/ml (R&D Systems, Abingdon, UK); Matrix metallopeptidase 9/Tissue inhibitor of metallopeptidase-1 complex (MMP-9/TIMP-1), MDD 0.0469 ng/ml (R&D Systems, Abingdon, UK); Plasminogen activator inhibitor-1/Tissue plasminogen activator complex (PAI-1/tPA), MDD 0.04 ngml (AssayPro, Missouri, US); Phospho-extracellular signal-related kinase (pERK), MDD 18.75 pg/ml (MyBioSource, San Diego, UK); Progranulin, MDD 0.17 ng/ml (R&D Systems, Abingdon, UK), S100 calcium-binding protein A4 (S100A4), MDD 0.225 ng/ml (Cusabio, Houston, US); Transforming growth factor beta-1 (TGFβ1), MDD 4.61 pg/ml (R&D Systems, Abingdon, UK); Thrombomodulin, MDD 7.82 pg/ml (R&D Systems, Abingdon, UK) and Tissue plasminogen activator (TPA), MDD 0.01 ng/ml (Abcam, Cambridge, UK).

**Point of care assays and investigations**

At recruitment, patient urine samples were collected prior to cytoscopic examination and evaluated using the point of care test (POCT) Nuclear Matrix Protein 22 (NMP22) (BladderChek, Alere, US), according to manufacturer’s instructions (MDD <10 U/ml were negative). Aution sticks 10EA, used for dipstick urinalysis, were interpreted using a PocketChem analyser (Arkray Inc, Japan).

**Osmolality**

Osmolality (mOsm) was determined using a Lӧser Micro-osmometer according to manufacturer’s instructions (Loser Messtechnik, Berlin, Germany).

**Total Urinary Protein (Bradford Assay)**

Total urinary protein levels (mg/ml) were determined, in triplicate, by Bradford assay (Pierce, Rockford, IL, USA) using a stock solution of BSA (Sigma) as standard (1 mg/ml). Patient urine samples (10 μl/patient), after centrifugation (1200 g, 10 minutes, 4^o^C), were mixed with Bradford reagent (1 ml) and allowed to stand for 5 minutes. The samples were read on a Hitachi Spectrophotometer (Model No. U-2800) at A_595_nm. Total urinary protein was determined using a BSA calibration chart.

[1] FitzGerald SP, Lamont J V., McConnell RI, Benchikh EO. Development of a high-throughput automated analyzer using biochip array technology. Clin Chem 2005;51:1165–76. https://doi.org/10.1373/clinchem.2005.049429.

**Supplementary 3 Urine Biomarkers**

| **Biomarker (Urine)** | **Control** | **Bladder Cancer** | **p value** | **AUROC** | **Sensitivity** | **Specificity** | **PPV (%)** | **NPV (%)** |
| --- | --- | --- | --- | --- | --- | --- | --- | --- |
| 80HdG (ng/ml) | 57.3 ± 40.4 (n=473) | 62.7 ± 40.9 (n=194) | 0.058 | 0.547 (0.500 - 0.594) | 0.560 (0.516 - 0.603) | 0.526 (0.459 - 0.598) | 74.2 | 32.9 |
| ACR | 4.9 ± 10.5 (n=473) | 7.7 ± 10.1 (n=199) | <0.001 | 0.628 (0.581 - 0.675) | 0.613 (0.571 - 0.655) | 0.598 (0.528 - 0.663) | 78.4 | 39.4 |
| BTA (U/ml) | 17.5 ± 30.2 (n=473) | 38.2 ± 42.0 (n=199) | <0.001 | 0.661 (0.615 - 0.707) | 0.674 (0.632 - 0.715) | 0.563 (0.492 - 0.633) | 78.6 | 42.1 |
| CK-18 (ng/ml) | 28.0 ± 84.9 (n=460) | 158.4 ± 463.8 (n=176) | <0.001 | 0.696 (0.650 - 0.742) | 0.674 (0.633 - 0.715) | 0.636 (0.568 - 0.705) | 82.9 | 42.7 |
| CK-20 (ng/ml) | 0.2 ± 0.1 (n=473) | 0.2 ± 0.2 (n=194) | 0.004 | 0.569 (0.522 - 0.617) | 0.507 (0.463 - 0.554) | 0.619 (0.546 - 0.686) | 76.4 | 34.0 |
| Clusterin (ng/ml) | 243.4 ± 324.7 (n=473) | 503.4 ± 564.7 (n=199) | <0.001 | 0.685 (0.641 - 0.730) | 0.658 (0.615 - 0.700) | 0.658 (0.593 - 0.724) | 82.1 | 44.7 |
| Creatinine (μmolL) | 8118.7 ± 5072.5 (n=473) | 10092.8 ± 6288.9 (n=199) | <0.001 | 0.600 (0.554 - 0.646) | 0.539 (0.495 - 0.584) | 0.608 (0.543 - 0.673) | 76.6 | 35.7 |
| CRP (mg/ml) | 1.4 ± 0.5 (n=473) | 1.5 ± 0.7 (n=199) | 0.157 | 0.535 (0.485 - 0.584) | 0.573 (0.529 - 0.617) | 0.482 (0.412 - 0.553) | 72.5 | 32.2 |
| CXCL16 (ng/ml) | 0.1 ± 0.3 (n=472) | 0.1 ± 0.3 (n=193) | <0.001 | 0.662 (0.615 - 0.709) | 0.633 (0.593 - 0.676) | 0.601 (0.528 - 0.668) | 79.5 | 40.1 |
| Cystatin B (ng/ml) | 8.9 ± 18.2 (n=473) | 20.0 ± 29.3 (n=199) | <0.001 | 0.641 (0.594 - 0.688) | 0.584 (0.539 - 0.628) | 0.633 (0.568 - 0.698) | 79.1 | 39.0 |
| Cystatin C (ng/ml) | 29.8 ± 43.5 (n=473) | 40.9 ± 40.3 (n=197) | <0.001 | 0.621 (0.576 - 0.666) | 0.569 (0.524 - 0.613) | 0.609 (0.538 - 0.675) | 77.7 | 37.0 |
| d-Dimer (ng/ml) | 49.3 ± 206.9 (n=473) | 215.4 ± 487.5 (n=199) | <0.001 | 0.657 (0.611 - 0.702) | 0.662 (0.619 - 0.704) | 0.593 (0.523 - 0.663) | 79.4 | 42.4 |
| EGF (pg/ml) | 8833.8 ± 11605.4 (n=473) | 15673.4 ± 19300.7 (n=197) | <0.001 | 0.616 (0.569 - 0.664) | 0.562 (0.518 - 0.605) | 0.614 (0.543 - 0.680) | 77.8 | 36.9 |
| FAS (pg/ml) | 25.1 ± 77.6 (n=473) | 53.4 ± 104.4 (n=199) | <0.001 | 0.626 (0.576 - 0.675) | 0.706 (0.666 - 0.746) | 0.523 (0.452 - 0.588) | 77.9 | 42.8 |
| IFNγ (pg/ml) | 2.0 ± 1.1 (n=473) | 2.0 ± 0.7 (n=199) | 0.001 | 0.528 (0.508 - 0.549) | 0.973 (0.958 - 0.985) | 0.085 (0.050 - 0.126) | 71.7 | 56.7 |
| IL-10 (pg/ml) | 1.1 ± 0.6 (n=473) | 1.1 ± 1.0 (n=199) | 0.386 | 0.509 (0.487 - 0.532) | 0.934 (0.911 - 0.958) | 0.085 (0.050 - 0.126) | 70.8 | 35.4 |
| IL-12p70 (pg/ml) | 3.4 ± 2.9 (n=472) | 3.6 ± 5.1 (n=199) | 0.018 | 0.447 (0.404 - 0.490) | 0.525 (0.481 - 0.574) | 0.392 (0.327 - 0.457) | 67.2 | 25.8 |
| IL-13 (pg/ml) | 6.4 ± 6.1 (n=472) | 8.1 ± 14.1 (n=199) | 0.019 | 0.551 (0.508 - 0.594) | 0.642 (0.597 - 0.686) | 0.462 (0.392 - 0.533) | 73.9 | 35.2 |
| IL-1α (pg/ml) | 4.5 ± 17.9 (n=473) | 23.6 ± 69.9 (n=199) | <0.001 | 0.628 (0.582 - 0.675) | 0.685 (0.643 - 0.727) | 0.538 (0.467 - 0.608) | 77.9 | 41.8 |
| IL-1β (pg/ml) | 14.6 ± 44.3 (n=473) | 26.3 ± 62.1 (n=199) | 0.001 | 0.570 (0.526 - 0.614) | 0.615 (0.571 - 0.660) | 0.497 (0.427 - 0.568) | 74.4 | 35.2 |
| IL-2 (pg/ml) | 4.7 ± 2.2 (n=473) | 4.9 ± 3.0 (n=199) | 0.562 | 0.504 (0.489 - 0.520) | 0.979 (0.964 - 0.992) | 0.040 (0.015 - 0.070) | 70.8 | 44.4 |
| IL23 (pg/ml) | 0.0 ± 0.0 (n=472) | 0.0 ± 0.0 (n=199) | 0.48 | 0.496 (0.483 - 0.508) | 0.017 (0.006 - 0.030) | 0.975 (0.950 - 0.995) | 61.5 | 29.5 |
| IL-3 (pg/ml) | 8.2 ± 4.2 (n=472) | 8.4 ± 5.1 (n=199) | 0.328 | 0.495 (0.485 - 0.504) | 0.998 (0.994 - 1.000) | 0.010 (0.000 - 0.025) | 70.5 | 66.7 |
| IL-4 (pg/ml) | 3.6 ± 1.6 (n=473) | 3.5 ± 1.1 (n=199) | 0.225 | 0.480 (0.447 - 0.513) | 0.167 (0.133 - 0.201) | 0.789 (0.729 - 0.844) | 65.3 | 28.5 |
| IL-6 (pg/ml) | 21.4 ± 96.1 (n=473) | 74.5 ± 186.8 (n=199) | <0.001 | 0.611 (0.563 - 0.659) | 0.550 (0.503 - 0.594) | 0.618 (0.548 - 0.683) | 77.4 | 36.6 |
| IL-7 (pg/ml) | 3.5 ± 3.3 (n=472) | 4.6 ± 3.1 (n=199) | <0.001 | 0.634 (0.588 - 0.679) | 0.648 (0.608 - 0.691) | 0.573 (0.508 - 0.638) | 78.3 | 40.7 |
| IL-8 (pg/ml) | 245.1 ± 464.3 (n=473) | 425.7 ± 556.5 (n=199) | <0.001 | 0.631 (0.586 - 0.677) | 0.586 (0.541 - 0.630) | 0.638 (0.573 - 0.709) | 79.4 | 39.3 |
| MCP-1 (pg/ml) | 135.5 ± 185.2 (n=473) | 207.5 ± 235.1 (n=199) | <0.001 | 0.637 (0.592 - 0.683) | 0.516 (0.471 - 0.560) | 0.704 (0.638 - 0.764) | 80.5 | 37.9 |
| Microalbumin (mg/l) | 33.6 ± 59.4 (n=473) | 69.1 ± 83.8 (n=199) | <0.001 | 0.677 (0.634 - 0.721) | 0.584 (0.539 - 0.628) | 0.704 (0.638 - 0.769) | 82.4 | 41.5 |
| Midkine (pg/ml) | 327.4 ± 521.5 (n=473) | 888.8 ± 1232.4 (n=199) | <0.001 | 0.655 (0.607 - 0.702) | 0.645 (0.603 - 0.689) | 0.603 (0.533 - 0.668) | 79.4 | 41.7 |
| MMP-9 (ng/ml) | 15.8 ± 39.6 (n=473) | 22.3 ± 53.9 (n=199) | 0.64 | 0.489 (0.439 - 0.539) | 0.643 (0.600 - 0.685) | 0.432 (0.367 - 0.503) | 72.9 | 33.7 |
| MMP-9/NGAL (ng/ml) | 4.6 ± 10.6 (n=473) | 7.3 ± 12.4 (n=199) | <0.001 | 0.603 (0.556 - 0.649) | 0.571 (0.528 - 0.615) | 0.573 (0.503 - 0.638) | 76.1 | 36.0 |
| MMP-9/TIMP-1 (ng/ml) | 0.6 ± 3.4 (n=473) | 2.2 ± 6.6 (n=199) | <0.001 | 0.625 (0.580 - 0.669) | 0.636 (0.594 - 0.681) | 0.563 (0.492 - 0.633) | 77.6 | 39.4 |
| NGAL (ng/ml) | 408.3 ± 501.8 (n=473) | 515.8 ± 593.0 (n=199) | 0.021 | 0.556 (0.508 - 0.604) | 0.600 (0.556 - 0.645) | 0.497 (0.427 - 0.563) | 74.0 | 34.4 |
| NMP22 (POC) | 18/473 (3.8%) | 39/199 (19.6%) | <0.001 | 0.579 (0.550-0.608) | 0.196 (0.141-0.256) | 0.962 (0.945-0.979) | 68.4 | 74.0 |
| NSE (ng/ml) | 0.6 ± 1.8 (n=473) | 6.5 ± 21.4 (n=199) | <0.001 | 0.678 (0.633 - 0.723) | 0.658 (0.613 - 0.700) | 0.618 (0.553 - 0.688) | 80.4 | 43.2 |
| Osmolality (mOsm) | 530.4 ± 231.6 (n=473) | 580.8 ± 188.7 (n=199) | 0.007 | 0.566 (0.522 - 0.611) | 0.488 (0.444 - 0.533) | 0.648 (0.583 - 0.714) | 76.7 | 34.8 |
| pERK (pg/ml) | 393.2 ± 335.2 (n=472) | 412.2 ± 367.4 (n=194) | 0.589 | 0.487 (0.439 - 0.534) | 0.519 (0.475 - 0.566) | 0.505 (0.433 - 0.577) | 71.8 | 30.2 |
| Progranulin (ng/ml) | 13.3 ± 10.3 (n=473) | 18.2 ± 18.3 (n=196) | <0.001 | 0.616 (0.570 - 0.662) | 0.543 (0.499 - 0.588) | 0.638 (0.571 - 0.704) | 78.4 | 36.7 |
| Protein (mg/ml) | 0.1 ± 0.3 (n=473) | 0.4 ± 0.7 (n=199) | <0.001 | 0.631 (0.584 - 0.679) | 0.615 (0.571 - 0.660) | 0.583 (0.513 - 0.653) | 77.8 | 38.9 |
| sIL-6R (ng/ml) | 0.6 ± 0.1 (n=473) | 0.6 ± 0.0 (n=199) | 0.489 | 0.503 (0.496 - 0.510) | 0.011 (0.002 - 0.021) | 0.995 (0.985 - 1.000) | 83.3 | 29.7 |
| sTNFRI (ng/ml) | 0.8 ± 0.8 (n=473) | 1.0 ± 0.8 (n=199) | <0.001 | 0.605 (0.559 - 0.651) | 0.664 (0.622 - 0.706) | 0.492 (0.422 - 0.563) | 75.7 | 38.1 |
| sTNRFII (ng/ml) | 1.7 ± 2.1 (n=473) | 1.9 ± 2.0 (n=199) | 0.02 | 0.557 (0.509 - 0.604) | 0.545 (0.503 - 0.592) | 0.553 (0.487 - 0.618) | 74.4 | 33.8 |
| TGFβ1 (pg/ml) | 39.3 ± 128.0 (n=473) | 118.0 ± 354.8 (n=199) | <0.001 | 0.627 (0.580 - 0.674) | 0.630 (0.586 - 0.674) | 0.548 (0.477 - 0.613) | 76.8 | 38.4 |
| Thrombomodulin (ng/ml) | 15.1 ± 10.1 (n=473) | 18.2 ± 10.3 (n=199) | <0.001 | 0.599 (0.554 - 0.644) | 0.571 (0.524 - 0.613) | 0.588 (0.518 - 0.653) | 76.7 | 36.6 |
| TNFα (pg/ml) | 4.3 ± 2.3 (n=473) | 5.7 ± 4.8 (n=199) | <0.001 | 0.575 (0.531 - 0.619) | 0.660 (0.617 - 0.702) | 0.442 (0.372 - 0.513) | 73.8 | 35.3 |
| TPA (ng/ml) | 1.6 ± 3.6 (n=473) | 2.9 ± 4.6 (n=197) | <0.001 | 0.636 (0.590 - 0.683) | 0.596 (0.550 - 0.638) | 0.650 (0.584 - 0.716) | 80.3 | 40.1 |
| VEGF (pg/ml) | 147.6 ± 209.2 (n=473) | 335.0 ± 526.2 (n=199) | <0.001 | 0.641 (0.594 - 0.689) | 0.643 (0.600 - 0.685) | 0.568 (0.497 - 0.638) | 77.9 | 40.1 |

8OHdG=8-hydroxy 2 deoxyguanosine; ACR=albumin creatinine ratio; BTA=bladder tumour antigen; CK=cytokeratin; CRP=C-reactive protein; CXCL16=chemokine (C-X-C motif) ligand 16 ;EGF=epithelial growth factor; FAS=tumour necrosis factor receptor superfamily member 6; IFNγ=interferon gamma; IL=interleukin; MCP-1=monocyte chemoattractant protein-1; MMP-9=matrix metallopeptidase-9; NGAL=neutrophil gelatinase-associated lipocalin; TIMP-1=tissue inhibitor metallopeptidase-1; NMP22=nuclear matrix protein 22; NSE=neuron specific enolase; pERK=phospho extracellular signal-related kinase; sIL-6R=soluble interleukin 6 receptor; sTNFR=soluble tumour necrosis factor receptor; TGFβ1=transforming growth factor β1; TNFα=tumour necrosis factor α; TPA=tissue plasminogen activator; VEGF=vascular endothelial growth factor

**Supplementary 4 Serum Biomarkers**

| **Biomarker (Serum)** | **Control** | **Bladder Cancer** | **p value** | **AUROC** | **Sensitivity** | **Specificity** | **PPV (%)** | **NPV (%)** |
| --- | --- | --- | --- | --- | --- | --- | --- | --- |
| CD44 (ng/ml) | 136.8 ± 58.9 (n=472) | 155.6 ± 75.5 (n=199) | 0.019 | 0.557 (0.507 - 0.608) | 0.644 (0.604 - 0.686) | 0.477 (0.407 - 0.548) | 74.5 | 36.1 |
| CEA (ng/ml) | 2.1 ± 1.9 (n=473) | 3.3 ± 11.8 (n=199) | 0.011 | 0.562 (0.516 - 0.609) | 0.573 (0.529 - 0.619) | 0.533 (0.462 - 0.598) | 74.5 | 34.4 |
| Cholesterol (mmol/l) | 4.8 ± 1.2 (n=459) | 4.5 ± 1.2 (n=191) | 0.013 | 0.562 (0.512 - 0.611) | 0.614 (0.569 - 0.660) | 0.513 (0.445 - 0.586) | 75.2 | 35.6 |
| CRP (mg/ml) | 3.3 ± 8.1 (n=458) | 3.9 ± 7.6 (n=190) | 0.001 | 0.582 (0.534 - 0.631) | 0.539 (0.493 - 0.583) | 0.616 (0.547 - 0.684) | 77.2 | 35.7 |
| Cystatin C (ng/ml) | 1.0 ± 0.3 (n=470) | 1.0 ± 0.3 (n=197) | 0.307 | 0.525 (0.475 - 0.575) | 0.638 (0.596 - 0.681) | 0.447 (0.376 - 0.513) | 73.3 | 34.1 |
| EGF (pg/ml) | 16.3 ± 17.5 (n=469) | 26.1 ± 27.0 (n=197) | <0.001 | 0.616 (0.568 - 0.663) | 0.499 (0.454 - 0.544) | 0.690 (0.624 - 0.756) | 79.3 | 36.7 |
| FABP-A (ng/ml) | 35.0 ± 23.9 (n=473) | 39.3 ± 31.7 (n=199) | 0.282 | 0.526 (0.478 - 0.574) | 0.548 (0.501 - 0.590) | 0.508 (0.437 - 0.573) | 72.5 | 32.1 |
| GROα (pg/ml) | 95.6 ± 101.5 (n=473) | 101.7 ± 90.8 (n=199) | 0.001 | 0.579 (0.533 - 0.625) | 0.507 (0.463 - 0.552) | 0.643 (0.578 - 0.709) | 77.2 | 35.5 |
| HAD (U/l) | 0.3 ± 0.5 (n=473) | 0.3 ± 0.7 (n=197) | 0.08 | 0.543 (0.497 - 0.588) | 0.427 (0.378 - 0.471) | 0.660 (0.594 - 0.726) | 75.1 | 32.4 |
| HDL (mmol/l) | 1.2 ± 0.4 (n=459) | 1.2 ± 0.4 (n=191) | 0.242 | 0.529 (0.479 - 0.579) | 0.573 (0.527 - 0.617) | 0.455 (0.382 - 0.524) | 71.7 | 30.7 |
| IFNγ (pg/ml) | 2.2 ± 2.6 (n=469) | 2.0 ± 0.8 (n=197) | 0.753 | 0.503 (0.487 - 0.518) | 0.041 (0.023 - 0.060) | 0.964 (0.934 - 0.985) | 73.1 | 29.7 |
| IL-10 (pg/ml) | 2.4 ± 21.5 (n=469) | 1.3 ± 1.8 (n=197) | 0.251 | 0.525 (0.483 - 0.566) | 0.405 (0.362 - 0.454) | 0.629 (0.563 - 0.696) | 72.2 | 30.8 |
| IL-18 (pg/ml) | 225.5 ± 434.3 (n=471) | 159.3 ± 291.3 (n=198) | 0.925 | 0.502 (0.459 - 0.546) | 0.573 (0.529 - 0.618) | 0.470 (0.399 - 0.535) | 72.0 | 31.6 |
| IL-1α (pg/ml) | 0.9 ± 1.1 (n=469) | 0.9 ± 0.2 (n=197) | 0.157 | 0.488 (0.470 - 0.506) | 0.032 (0.017 - 0.049) | 0.949 (0.919 - 0.975) | 60.0 | 29.2 |
| IL-1β (pg/ml) | 1.4 ± 1.2 (n=469) | 1.2 ± 0.2 (n=197) | 0.708 | 0.504 (0.485 - 0.523) | 0.062 (0.043 - 0.085) | 0.944 (0.909 - 0.975) | 72.5 | 29.7 |
| IL-2 (pg/ml) | 5.2 ± 4.7 (n=469) | 4.5 ± 1.0 (n=197) | 0.075 | 0.518 (0.501 - 0.535) | 0.070 (0.049 - 0.094) | 0.964 (0.934 - 0.990) | 82.5 | 30.4 |
| IL-4 (pg/ml) | 3.6 ± 2.4 (n=469) | 3.3 ± 0.7 (n=197) | 0.073 | 0.530 (0.499 - 0.560) | 0.198 (0.164 - 0.235) | 0.858 (0.807 - 0.904) | 76.9 | 31.0 |
| IL-6 (pg/ml) | 4.1 ± 7.6 (n=469) | 4.2 ± 4.8 (n=197) | 0.007 | 0.566 (0.519 - 0.614) | 0.618 (0.574 - 0.663) | 0.523 (0.457 - 0.594) | 75.5 | 36.5 |
| IL-8 (pg/ml) | 14.0 ± 12.3 (n=469) | 14.7 ± 10.8 (n=197) | 0.388 | 0.479 (0.430 - 0.528) | 0.505 (0.458 - 0.550) | 0.523 (0.452 - 0.594) | 71.6 | 30.7 |
| LASP-1 (pg/ml) | 394.5 ± 300.8 (n=473) | 501.1 ± 552.9 (n=199) | 0.026 | 0.554 (0.506 - 0.602) | 0.526 (0.482 - 0.573) | 0.558 (0.487 - 0.623) | 73.9 | 33.1 |
| LDL (mmol/l) | 2.8 ± 1.0 (n=459) | 2.6 ± 1.1 (n=191) | 0.018 | 0.559 (0.509 - 0.609) | 0.595 (0.551 - 0.638) | 0.534 (0.461 - 0.607) | 75.4 | 35.4 |
| M2PK (ng/ml) | 32.9 ± 59.3 (n=472) | 31.6 ± 16.9 (n=198) | 0.83 | 0.495 (0.446 - 0.544) | 0.477 (0.430 - 0.521) | 0.535 (0.465 - 0.606) | 71.0 | 30.0 |
| M30 (U/l) | 175.2 ± 139.7 (n=473) | 170.6 ± 137.2 (n=199) | 0.984 | 0.499 (0.452 - 0.547) | 0.560 (0.516 - 0.605) | 0.477 (0.407 - 0.553) | 71.8 | 31.4 |
| MCP-1 (pg/ml) | 387.7 ± 131.3 (n=469) | 400.8 ± 148.6 (n=197) | 0.308 | 0.525 (0.476 - 0.574) | 0.429 (0.382 - 0.473) | 0.629 (0.563 - 0.701) | 73.4 | 31.6 |
| PAI-1/tPA (ng/ml) | 8.2 ± 7.0 (n=473) | 12.6 ± 9.8 (n=199) | <0.001 | 0.667 (0.621 - 0.713) | 0.672 (0.630 - 0.715) | 0.593 (0.523 - 0.658) | 79.7 | 43.2 |
| Prolactin (mlU/l) | 260.9 ± 248.0 (n=468) | 227.9 ± 162.0 (n=196) | 0.001 | 0.581 (0.534 - 0.629) | 0.637 (0.592 - 0.679) | 0.526 (0.454 - 0.597) | 76.2 | 37.7 |
| PSA/tPSA (ng/ml) | 2.4 ± 4.1 (n=473) | 2.3 ± 4.5 (n=199) | 0.533 | 0.515 (0.469 - 0.562) | 0.450 (0.406 - 0.495) | 0.618 (0.548 - 0.683) | 73.7 | 32.1 |
| S100A4 (ng/ml) | 54.3 ± 34.8 (n=473) | 61.7 ± 45.0 (n=199) | 0.145 | 0.536 (0.487 - 0.585) | 0.588 (0.543 - 0.634) | 0.518 (0.447 - 0.588) | 74.3 | 34.6 |
| sIL-2Ra (ng/ml) | 0.1 ± 0.1 (n=473) | 0.1 ± 0.1 (n=199) | 0.037 | 0.530 (0.500 - 0.561) | 0.882 (0.852 - 0.911) | 0.176 (0.126 - 0.231) | 71.8 | 38.5 |
| TNFα (pg/ml) | 4.3 ± 2.8 (n=469) | 4.0 ± 0.9 (n=197) | 0.818 | 0.505 (0.462 - 0.549) | 0.565 (0.522 - 0.610) | 0.487 (0.416 - 0.553) | 72.4 | 32.0 |
| Triglycerides (mmol/l) | 1.7 ± 1.0 (n=459) | 1.4 ± 0.7 (n=191) | 0.003 | 0.573 (0.525 - 0.621) | 0.588 (0.542 - 0.634) | 0.508 (0.435 - 0.576) | 74.2 | 33.9 |
| VEGF (pg/ml) | 242.5 ± 161.9 (n=469) | 293.1 ± 202.9 (n=197) | 0.002 | 0.575 (0.527 - 0.622) | 0.525 (0.478 - 0.569) | 0.584 (0.513 - 0.650) | 75.0 | 34.0 |

CD44=cluster of differentiation 44 ; CEA=carcinoembryonic antigen; CRP=C-reactive protein; EGF=epidermal growth factor; FABPA=fatty acid binding protein adipocyte; CXCL1/GROalpha=C-X-C ligand 1 motif/growth regulated alpha protein; HAD=hyaluronic acid; HDL=high density lipoprotein; IFNγ=interferon gamma; IL=interleukin; LASP-1=LIM and SH3 domain; LDL=low density lipoprotein; M2-PK=muscle type-2 pyruvate kinase; M30= caspase-cleaved CK-18 fragments; MCP-1= MCP-1=monocyte chemoattractant protein-1; PAI-1/tPA=plasminogen associated inhibitor-1/tissue plasminogen activator; PSA=prostate serum antigen; tPSA=total prostate serum antigen; S100A4=S100 calcium-binding protein A4; sIL-2Ra=soluble interleukin 2 receptor alpha; TNFα=tumour necrosis factor alpha; VEGF=vascular endothelial growth factor
